# The most common diagnoses in primary care, and changes over time, in the total population of Stockholm, Sweden

**DOI:** 10.1186/s12875-025-02938-3

**Published:** 2025-08-01

**Authors:** Per Wändell, Gunnar Ljunggren, Axel C. Carlsson

**Affiliations:** 1 Division of Family Medicine and Primary Care, Department of Neurobiology, Care Sciences and Society, Karolinska Institutet, Alfred Nobels allé 23, Huddinge, S-141 83 Sweden; 2 Center for Primary Health Care Research, Lund University, Malmö, Sweden; 3 Academic Primary Health Care Centre, Stockholm Region, Stockholm, Sweden

**Keywords:** Primary care, Sex, Women in family medicine, Caregivers, Epidemiology, Quantitative Research/Study, Administrative database

## Abstract

**Objective:**

Primary care is the base in many health care systems, and to identify the most registered diagnoses in primary care is a way to identify the overall health care use and needs in society. We estimated the rates of the 30 most common diagnoses in primary health care and their male to female ratio.

**Research design and methods:**

This was a study including inhabitants 18 years and older out of all 2.3 million inhabitants living in Region Stockholm, Sweden. Data on all healthcare appointments from primary care during 2019–2021 were extracted from the Stockholm County Council data warehouse known as VAL. Primary care data were analyzed by underlying population and age. In 2019, only physical visits were available, but during 2021 digital visits were included. For the specific diagnoses, physical and digital visits were merged.

**Results:**

The five most common diagnoses in primary care were: essential hypertension (I10), myalgia (M79), type 2 diabetes (E11), dorsalgia (M54), and pain in joint (M25). The female-to-male ratios were higher for 27 of the 30 most common diagnoses, for example stress reaction (F43), malaise and fatigue (R53), and headache (R51). Chronic ischaemic heart disease (I25), Type 2 diabetes (E11), and Atrial fibrillation (I48) were more common in men.

**Conclusions:**

Most of the common diagnoses in primary care are more often registered in women than in men. The higher presence of diagnoses of pain and mental illness seems to mirror the higher sick leave among women in recent years.

**Supplementary Information:**

The online version contains supplementary material available at 10.1186/s12875-025-02938-3.

## Introduction

Primary care has an essential role in the health care system in most countries [1]. A higher accessibility to primary health care physicians contributes not only to improvement in public health and but also to lower total health care costs [2, 3].

Swedish health care is to a large proportion publicly funded by the county councils [4]. In Sweden, primary care has traditionally had a weaker role in the health care system compared to in several other countries, e.g., in the UK [5]. The goal according to the Swedish Health and Medical Services Act from 1982 is that all citizens should have a good health and equal access to health care [6]. Yet, there are vast differences in health and access to primary care depending on socio-economic status and many socio-economic factors may affect equity in care negatively. For example, since the 1990-ies the Swedish health care sector has undergone privatization [7]. Furthermore, digital visits, or telemedicine, has become more common in recent years and their availability are also limited in vulnerable populations [8, 9].

The development of electronic medical records and administrative databases have facilitated studies [10], not only on the prevalence and incidence of diseases in large populations in entire regions [11, 12], but also on the impact of different changes in the health care system [6]. The pattern of diseases might differ between countries, and a review concluded, that “There are differences between clinician-reported and patient-reported reasons for visits to primary care, as well as differences between developed and developing countries” [13]. We have earlier reported on the 30 most common diagnoses in primary care in Region Stockholm 2009–2011 [14]. The study has been cited extensively and many similar studies have been conducted since [13].

Accordingly, the aim of the present study was to follow visits in primary health care over time, in general and by diagnoses as well as their male-to female ratio, but also to compare the pattern with the patterns in the new studies from other countries and from our earlier study.

## Methods and study population

The study population included all inhabitants aged 18 years and above (*n* = 1 854 344) living in Region Stockholm January 1, 2019. The population of Region Stockholm represents around more than one-fourth of Sweden’s entire population. This area of Sweden includes the capital city of Stockholm and several other cities and towns, as well as large rural areas and a sparsely populated archipelago. The Region Stockholm Council is responsible for financing primary and secondary health care through taxes. Primary health care accounts for around half of all appointments to physicians in ambulatory care. Most hospital services are provided by county-owned facilities, while private care providers manage many primary care and specialist open care units. However, during the last decades, much of the primary care and acute hospital care services have been outsourced to private providers. Private providers are in contractual agreements with the Region and are legally obliged to record diagnoses and report them to the authorities in the same way as public providers [15]. Apart from very few private clinics that operate without subsidies in Stockholm, all appointments and diagnoses are recorded and stored in VAL, the central regional data warehouse. The diagnoses in the VAL register are coded by the ICD-10 codes. Acute and chronic diagnoses are often mixed, and acute conditions may be the start of a chronic condition, but it is not so easy to separate registered acute from chronic diagnoses. A maximum of 8 ICD-10 diagnoses can be registered for each consultation in primary care. Approximately 95% of all physician appointments in primary care have at least one diagnosis being recorded and stored in VAL Furthermore, VAL has more than 99% coverage of inpatient care [16]. Thus, VAL contains data from a large, unselected population that can be utilized for epidemiological research [12].

For this study, data were extracted from VAL on all physician appointments made in primary care in the years 2019–2021. Unlike in 2019, in 2021, digital appointments were integrated in the care and also included. Inhabitants 18 years of age and older were included. We used the ICD-10 codes on group levels (one letter and the first two digits).

The VAL database has been used for about 20 years for research purposes, and has recently been used to study different conditions, including multimorbidity [17]. As an indication for its accuracy and validity, VAL is used by the county for updating the National Patient Register kept by the Swedish National Board of Health and Welfare (NBHW), as well as the annual benchmarking reports of the NBWH and the Swedish Association of Local Authorities and Regions [18].

The 30 most common diagnostic groups recorded during 2019, and cumulatively across 2019–2021, were identified. Female-to-male visit ratios were calculated for each diagnostic group to examine sex-based differences in care-seeking patterns. Additionally, the five most frequent diagnoses overall were highlighted, and their distribution by age groups was described. To further explore sex differences, the age distribution for the five diagnoses with the highest female-to-male ratios, and the three diagnoses with the lowest ratios, respectively, were examined. Additional diagnoses with notable sex differences but not included in the tables, were also noted in the text.

### Statistical methods

Standard statistics such as numbers and proportions were used to describe the study population. Analyses were performed by sex, and we also included female-to-male ratios, using the visits and registered diagnoses in the current sex and age group. Statistical analyses were performed in the SAS software, version 9.3 (SAS Institute Inc., Cary, NC).

## Results

The population in Region Stockholm January 1, 2019, 18 years and older amounted to 1 854 344 inhabitants, 933 981 women and 920 363 men (Table 1). The number of physical visits to physicians were 2 982 128 during 2019, and 2 158 941 during 2021. The number of digital visits during 2021 were 408 490. The average number of appointments in primary health care in Stockholm County per inhabitant (only physical visits) was 1.61 during 2019 (1.89 for women and 1.32 for men) and 1.16 during 2021 (1.38 for women and 0.95 for men). The number of digital visits was 0.22 in 2021 (0.28 for women and 0.16 for men), and for merged physical and digital visits 1.38 during 2021 (1.66 for women, and 1.11 for men, Supplementary Table S1).

**Table 1:** Age distribution in the population in region Stockholm January 1, 2019, the number of visits (with percentages) to physicians in primary care (PC) 2019 and 2021, with digital visits also included for 2021. No digital visits were recorded in 2019, and only very low numbers in 2021

	Population 2019 (*N* and %)			PC physical visits 2019 (*N* and %)			PC physical visits 2021 (*N* and %)			PC digital visits 2021 (*N* and %)			PC physical and digital visits 2021 (numbers)		
Age 2019	Sex			Sex			Sex			Sex			Sex		
(years)	Women	Men	Total	Women	Men	Total	Women	Men	Total	Women	Men	Total	Women	Men	Total
18–44	429 175 (46%)	446 143 (48%)	875 318 (47%)	597 466 (34%)	353 614 (29%)	951 080 (32%)	442 822 (34%)	266 082 (31%)	708 904 (33%)	158 394 (61%)	84 703 (58%)	243 097 (60%)	601 216 (39%)	350 785 (34%)	952 001 (37%)
45–64	289 466 (31%)	294 875 (32%)	584 341 (32%)	534 027 (30%)	371 900 (31%)	905 927 (30%)	414 510 (32%)	299 427 (34%)	713 937 (33%)	81 876 (31%)	47 728 (32%)	129 604 (32%)	496 386 (32%)	347 155 (34%)	843 541 (33%)
65–79	152 707 (16%)	139 098 (15%)	291 805 (16%)	395 347 (22%)	327 579 (27%)	722 926 (24%)	298 997 (23%)	226 245 (26%)	525 242 (24%)	17 653 (7%)	12 621 (9%)	30 274 (7%)	316 650 (20%)	238 866 (23%)	555 516 (22%)
80-w	62 633 (7%)	40 247 (4%)	102 880 (6%)	238 832 (14%)	163 363 (13%)	402 195 (13%)	132 227 (10%)	78 631 (9%)	210 858 (10%)	3 414 (1%)	2 101 (1%)	5 515 (1%)	135 641 (9%)	80 732 (8%)	216 373 (8%)
Total	933 981 (100%)	920 363 (100%)	1 854 344 (100%)	1 765 672 (100%)	1 216 456 (100%)	2 982 128 (100%)	1 288 556 (100%)	870 385 (100%)	2 158 941 (100%)	261 337 (100%)	147 153 (100%)	408 490 (100%)	1 549 893 (100%)	1 017 538 (100%)	2 567 431 (100%)

The 30 most common groups of diagnoses in 2019, and 2019–2021, are shown in Tables 2 and 3, where also the female-to-male ratio is shown. The female-to-male ratio was higher than one in 27 out of the 30 most common diagnoses, with the exceptions being chronic ischaemic heart disease (I25), type 2 diabetes (E11), and atrial fibrillation (I48). The five most common diagnoses are listed in Table 4, i.e. essential hypertension (I10), myalgia (M79), type 2 diabetes (E11), dorsalgia (M54), and pain in joint (M25). These five diagnoses include two diagnoses that were more common among elderly, i.e. essential hypertension (I10), and type 2 diabetes (E11), while the other three were more common among younger and middle-aged groups.

**Table 2:** Subjects with rank 1–15 of the most common diagnosis (with numbers and percentage of the population) during 2019 and 2019–2021 among the population January 1, 2019, in region Stockholm in primary care (PC), and with female to male ratio (F/M ratio) 2019, ranked after the visits for 2019

2019						2019–2021				
Rank	ICD code	ICD Diagnosis	PC diagnosis	Rate of population	F/M ratio	Rank	ICD code	ICD Diagnosis	PC diagnosis	Rate of population
1	I10	Essential hypertension	215,243	11.6%	1.08	1	I10	Essential hypertension	323,799	17.5%
2	M79	Myalgia	77,134	4.2%	1.71	2	M79	Myalgia	190,521	10.3%
3	E11	Type 2 diabetes	72,923	3.9%	0.73	3	M54	Dorsalgia	169,750	5.4%
4	M54	Dorsalgia	72,460	3.9%	1.44	4	M25	Paint in joint	161,044	9.2%
5	M25	Paint in joint	64,991	3.5%	1.46	5	J06	Upper respiratory inf.	151,705	8.7%
6	F41	Anxiety	64,870	3.5%	2.18	6	R10	Pain in the abdomen	145,546	7.1%
7	E78	Dyslipidaemia	60,236	3.2%	0.94	7	R52	Pain	132,291	5.2%
8	R10	Pain in the abdomen	57,676	3.1%	1.89	8	F41	Anxiety	131,705	7.8%
9	R52	Pain	57,222	3.1%	1.68	9	R53	Malaise and fatigue	124,190	7.1%
10	G47	Sleep disorders	55,647	3.0%	1.56	10	G47	Sleep disorders	109,648	5.9%
11	E03	Other hypothyroidism	51,561	2.8%	5.72	11	R23	Other skin changes	106,273	4.5%
12	R53	Malaise and fatigue	49,517	2.7%	2.41	12	E11	Type 2 diabetes	100,614	6.7%
13	R23	Other skin changes	41,947	2.3%	1.54	13	R05	Coughing	96,375	5.7%
14	J06	Upper respiratory inf.	41,613	2.2%	1.72	14	E78	Dyslipidaemia	95,634	8.2%
15	J45	Asthma	38,086	2.1%	1.88	15	N30	Cystitis	83,429	4.1%

**Table 3:** Subjects with rank 16–30 of the most common diagnosis (with numbers and percentage of the population) during 2019 and 2019–2021 among the population January 1, 2019, in region Stockholm in primary care (PC), and with female to male ratio (F/M ratio) 2019, ranked after the visits for 2019

2019						2019–2021				
Rank	ICD code	ICD Diagnosis	PC diagnosis	Rate of population	F/M ratio	Rank	ICD code	ICD Diagnosis	PC diagnosis	Rate of population
16	I48	Atrial fibrillation	36,727	2.0%	0.79	16	E03	Other hypothyroidism	82,701	3.1%
17	F43	Stress reaction	34,161	1.8%	3.03	17	F43	Stress reaction	81,423	4.4%
18	R05	Coughing	33,180	1.8%	1.43	18	J45	Asthma	76,538	5.2%
19	N30	Cystitis	32,024	1.7%	7.47	19	R22	Local swelling of the skin	62,640	4.5%
20	E66	Obesity	27,436	1.5%	1.38	20	R42	Dizziness	61,166	2.6%
21	R22	Local swelling of the skin	26,761	1.4%	1.86	21	F32	Depression	58,406	3.4%
22	L30	Other dermatitis	24,137	1.3%	1.45	22	I48	Atrial fibrillation	57,084	3.0%
23	F32	Depression	23,899	1.3%	1.81	23	R07	Pain in throat and chest	56,598	3.1%
24	R42	Dizziness	23,893	1.3%	2.15	24	R51	Headache	55,262	3.3%
25	U07	Uncertain etiology	23,867	1.3%	1.36	25	L30	Other dermatitis	54,953	2.7%
26	J30	Vasomotor and allergic rhinitis	21,516	1.2%	1.43	26	R06	Abnormalities of breathing	51,860	2.7%
27	R51	Headache	21,479	1.2%	2.23	27	J30	Vasomotor and allergic rhinitis	50,320	3.0%
28	R06	Abnormalities of breathing	21,117	1.1%	1.19	28	B34	Viral infection of unspecified site	50,310	2.8%
29	R21	Rash	21,082	1.1%	1.54	29	R21	Rash	49,988	2.7%
30	I25	Chronic ischaemic heart disease	21,033	1.1%	0.42	30	U07	Uncertain etiology	49,441	1.8%

**Table 4:** Age-distribution of the top five groups of most common diagnoses during 2019 of the population in region Stockholm January 1, 2019, with percentage in each age-group for each diagnosis

	Essential hypertension (I10)		Myalgia (M79)		Type 2 diabetes (E11)		Dorsalgia (M54)		Paint in joint (M25)	
Age 2019	Numbers	(%)	Numbers	(%)	Numbers	(%)	Numbers	(%)	Numbers	(%)
(years)										
18–44	9811	4.6	23,755	30.8	4006	5.5	23,500	32.4	16,699	25.7
45–64	72,130	33.5	30,974	40.2	26,278	36.0	27,978	38.6	27,867	42.9
65–79	96,951	45.0	17,169	22.3	32,805	45.2	15,549	31.5	16,037	24.7
80+	36,351	16.9	5236	6.8	9834	13.5	5433	7.5	4388	6.7
Total	215,243		77,134		72,923		72,460		64,991	

Tables 5 and 6 includes the age-distribution of the five diagnoses with the highest female-to-male ratio, and the three diagnoses with the lowest ratios, respectively. Besides these top diagnoses, dizziness (R42) and anxiety (F41) showed a high female-to-male ratio (data not shown in tables).

**Table 5:** Age-distribution of occurrence for each sex of the five diagnoses with highest female to male ratio in 2019 among the 30 most common diagnoses irrespective of the size of the groups

	Cystitis (N30)			Hypo-thyroidism (E03)			Stress reaction (F43)			Malaise and fatigue (R53)			Headache (R51)		
Age group	Women	Men	Ratio	Women	Men	Ratio	Women	Men	Ratio	Women	Men	Ratio	Women	Men	Ratio
18–44	11,109	670	16.58	10,666	1583	6.74	13,470	4636	2.91	16,630	5679	2.93	7467	3201	2.33
45–64	8589	1018	8.44	14,814	2540	5.83	11,182	3547	3.15	11,390	4885	2.33	4948	2370	2.09
65–79	6250	1510	4.14	13,014	2507	5.19	869	246	3.53	4761	2791	1.71	1947	923	2.11
80-w	2293	585	3.92	5397	1040	5.19	162	49	3.31	2235	1146	1.95	458	165	2.78
Total	28 241	3 783	7.47	43 891	7 670	5.72	25 683	8 478	3.03	35 016	14 501	2.41	14 820	6 659	2.23

**Table 6:** Age-distribution of the three diagnoses with lowest female to male ratio in 2019 among the 30 most common diagnoses

	Chronic ischaemic heart disease (I25)			Type 2 diabetes (E11)			Atrial fibrillation (I48)		
Age group (years)	Women	Men	Ratio	Women	Men	Ratio	Women	Men	Ratio
18–44	30	140	0.21	1681	2325	0.72	46	186	0.25
45–64	913	3910	0.23	10,100	16,178	0.62	1215	2884	0.42
65–79	2934	7663	0.38	13,657	19,148	0.71	7253	10,974	0.66
80+	2293	3150	0.73	5263	4571	1.15	7710	6459	1.19
Total	6170	14 863	0.42	30 701	42 222	0.73	16 224	20 503	0.79

## Discussion

In primary care in Region Stockholm, the five most common diagnoses included chronic diagnoses, hypertension and type 2 diabetes were as expected to be common, but also several pain syndromes were in the top. Only three out of the top 30 diagnoses were more common in men, namely chronic ischaemic heart disease, type 2 diabetes, and atrial fibrillation.

### Comparisons with earlier studies

Furthermore, some of the diagnoses in the present study were not noted among the 30 most common in the earlier study, such as stress reaction and headache. Among the five diagnoses with the highest female-to-male ratio, were also stress reaction, malaise and fatigue, and headache. Chronic pain is common in the population, being present in around 6% [19], and being often also among the elderly [20]. Pain and mental illness are common conditions, and are also connected in a bidirectional relationship [21]. The increased sick leave in Sweden during the first half of the 2010 decade, is mainly driven by psychiatric diagnoses according to the Swedish Insurance Fund, especially among women [22], mirrored by many of the top diagnoses and the high female-to-male ratio in the present study.

### Potential mechanisms for our findings

There are some potential explanations for the increase in stress-related disorders, and one important is that the workload has increased in the healthcare sector, without increasing funding. Even if this has been discussed much in Sweden, the exact explanations are not known.

Compared to results from the earlier mentioned review [13], many of the diagnoses found in developed countries were also found in our study, such as essential hypertension, dorsalgia or back pain, anxiety and depression. Upper respiratory infections were not listed in 2021, most certainly owing to the Covid-19 pandemic, while both upper respiratory infections and coughing were rather common when looking at the years 2019–2021. In the review, patient-reported reasons for visits were also reported, where symptomatic conditions were most reported. Interestingly, in our study symptomatic diagnoses were very common, rather than the more established chronic disease-diagnoses, such as hypertension, diabetes, hypothyroidism or asthma. This really seems to reflect primary care as the first line of health care.

The overall diagnosis pattern reflects the complex situation in primary care, with demands to take care of both acute situations as well as chronic disorders, such as hypertension, diabetes, and asthma [12]. Diagnostic accuracy may also vary, depending on the disorder and the diagnostic criteria. While correct diagnoses in most cases lead to appropriate management, the opposite is also true, i.e. incorrect diagnoses in many cases may lead to inappropriate management [23]. Yet, all patients are not easily diagnosed, and some individuals may need considerable counseling in order to obtain sufficient information to determine the most appropriate diagnosis or diagnoses. Challenging patients include individuals on long-term sick-leave, individuals exposed to domestic violence, and individuals suffering from treatment-resistant depression. Furthermore, multimorbidity is common, especially among the elderly [17]. However, multimorbidity was not within the scope of this specific study and has been assessed earlier in detail with this data [17]. Sometimes we see vague diagnoses or symptom diagnoses registered in primary care, instead of a more specific diagnosis, which could be registered later, or even be difficult to identify. It is also important to emphasize, that the frequency and the completeness of diagnoses may vary, both between individual physicians, and also between different primary health care centres [24]. The applicability of our method may also vary in different settings or countries. In Sweden, primary care has a weaker role in the health care system, compared to for example in the UK [5]. There is a variability in the number of inhabitants per general practitioner (GP) and in the extent to which patients relate to individual physicians, with lower rates in Sweden compared to other OECD countries [4]. The reimbursement system and mode of payment differs, and the use of specialist services varies accordingly [25]. Reimbursements systems may certainly affect both the number of visits, and the numbers of the recorded diagnoses. In Sweden, different models were introduced around 2010 in different parts of the country, and the model in Region Stockholm focused on “pay-per-visit” [26]. This changed the care seeking pattern to primary health care, with a higher proportion of acute visits, mostly upper respiratory infections, and also to “visit fragmentation”, i.e. more visits with shorter time instead of handling different problems at the same time during longer consultations [27]. However, the “pay-per-visit” model was scrapped in Region Stockholm in favour of a reimbursement model based firstly on the number of individuals listed at the primary health care centre, secondly on registered diagnoses and morbidity according to the Adjusted Clinical Groups (ACG) system [28], and thirdly the socio-economic index CNI [29]. This reimbursement model was also used during the years of the present study. The mean number of outpatient contacts in Sweden 2009 was 2.8 per person and year, to be compared with 6.2 in EU, and between 2.8 and 4.4 in the other Nordic countries [4]. The number of appointments with a GP at the primary health care centres (PHCC) was 1.5 per person and year in Sweden as a whole, to be compared to 1.6 in the present study in Stockholm County. The gatekeeping functions of GPs serves to keep queues to specialist care shorter and more relevant than without the sorting that primary care assists with, and making the health care expenses lower [30]. All these factors could also affect the recording of diagnoses. Furthermore, the systems of health care delivery are continuously changing in most countries, why patterns may differ both between countries and regions, and over time. Additionally, diseases and disease patterns vary over time, and digital meetings have been introduced, making recent data of value in comparison to earlier and future studies.

The sex difference pattern shows a preponderance for visits by women in general and for most diagnoses. The three diagnoses being more common in men compared to women are well established. Diabetes has been more common among men in Sweden since many years [31], and it is also well established that cardiovascular events including coronary heart disease is more common in men [32]. Furthermore, even if a registered diagnosis of hypertension is more common among women, in population studies undetected hypertension is more common in men, and hypertension affects men earlier in life compared to women [33].

In this study we merged physical and digital visits. It would also be of interest to look at the digital visits during 2021, and the development of this emerging part of primary care over time. Telemedicine contacts are not determined if they are associated with factors associated with high health care needs [8], and factors shown to be associated with a higher use of telemedicine contacts were younger age, higher educational attainment, higher income and being born in Sweden. The digital care needs to be assessed in detail. It remains to be shown how digital visits could be a complement to ordinary care and when they may replace physical visits without leading to a higher number of consultations as well as if the quality of health care could be affected. However, this is beyond the scope of the present study.

Another concern is the division into acute or chronic diagnoses. For some types of diagnoses this might be easy, but for others not. Pain diagnoses might be acute or chronic, and to be able to see that, other analyses are of importance. Besides, even if a diagnosis of an acute condition such as stroke is recorded when the condition occurs, the diagnosis sometimes tends to be set also later. Thus, we decided not to try to sub-divide.

In our earlier study of the 30 most common diagnoses, the age-group 0–17 years was included, which is why the pattern in the present study is expected to be slightly different, with an expected lower rate of some acute respiratory diagnoses. Besides, 2019 was the year prior to the Covid-19 pandemic, while 2021 was during the pandemic phase, and the number of visits in primary care per capita was lower during the pandemic. Additionally, digital visits were available during 2021.

*Limitations and strengths*.

This study has some limitations. The diagnoses are those registered in the electronic medical records, with a high number of symptom diagnoses. However, the situation in primary care, with people seeking care in early phases of diseases and disorders, and the need of further diagnostic procedures, makes the use of symptom diagnoses warranted and necessary in many cases, rather than to precede the diagnostic process and a premature diagnosis before the chronic condition has been correctly verified. The diagnostic accuracy is shifting between PHCCs but also over time, and we lack knowledge of how diagnostic criteria are followed in clinical practice. The use of the diagnosis “non-specified type of diabetes” is one example of the vagueness, even if one might suspect that most of these cases are type 2 diabetes. However, we have chosen to show the original coding instead of for instance merging “diabetes mellitus, not specified” into “type 2 diabetes mellitus”. Both under- and over-diagnostics are probably at hand [34]. The found prevalence of certain diagnoses seem rather low, e.g. of diabetes [35], and of depression [11, 36]. Furthermore, some diagnoses from hospitals are not recorded in primary care, even if the patients visit primary care after discharge from hospitals [37]. As regards the terms of gender or sex, we have used sex as found in the register, as self-reported gender other than the registered sex is not found in the database. Finally, the results from Stockholm County could not be valid as a proxy for national Swedish data. The population in Stockholm County is somewhat younger, has a higher educational level and higher mean income (according to Statistics Sweden). The reimbursement system which has changed over time could also affect the panorama of diagnoses.

## Conclusions

The pattern of diagnoses in primary care is complex. Even if the pattern over time in general seems stable with a higher female to male ratio of almost all diagnoses, some differences were found in the present study compared to a prior study conducted in Stockholm County, i.e. with higher presence of psychiatric and pain syndromes, also seemingly reflecting the higher sick leave among women in recent years. Increasingly more patients are transferred from specialist care to primary care, leading to changes in the pattern of diagnoses in primary health care over time important to follow. Furthermore, with the present trend in Sweden to increase the care close to patients, it is important to monitor the health of the population as mirrored by the situation in primary care over time.

## Supplementary Information

Supplementary Material 1.

## Data Availability

Interested parties can contact the corresponding author for potential collaborations. Furthermore, research data from the VAL database can be obtained after ethical approval by contacting the regional Centre for health data, email: halsodata.rst@regionstockholm.se.

## References

[1] Caley M. Remember Barbara starfield: primary care is the health system’s bedrock. BMJ. 2013;347:f4627. 23900532 10.1136/bmj.f4627

[2] Engstrom S, Foldevi M, Borgquist L. Is general practice effective? A systematic literature review. Scand J Prim Health Care. 2001;19(2):131–44. 11482415 10.1080/028134301750235394

[3] Starfield B. Is primary care essential? Lancet. 1994;344(8930):1129–33. 7934497 10.1016/s0140-6736(94)90634-3

[4] Anell A, Glenngard AH, Merkur S. Sweden health system review. Health Syst Transit. 2012;14(5):1–159. 22894859

[5] Forrest CB. Primary care in the united states: primary care gatekeeping and referrals: effective filter or failed experiment? BMJ. 2003;326(7391):692–5. 12663407 10.1136/bmj.326.7391.692 PMC152368

[6] Beckman A, Anell A. Changes in health care utilisation following a reform involving choice and privatisation in Swedish primary care: a five-year follow-up of GP-visits. BMC Health Serv Res. 2013;13:452. 24171894 10.1186/1472-6963-13-452 PMC4228471

[7] Blomqvist P. The choice revolution: privatization of Swedish welfare services in the 1990s. Social Policy Adm. 2004;38(2):139–55.

[8] Dahlgren C, Dackehag M, Wandell P, Rehnberg C. Determinants for use of direct-to-consumer telemedicine consultations in primary healthcare-a registry based total population study from stockholm, Sweden. BMC Fam Pract. 2021;22(1):133. 34172009 10.1186/s12875-021-01481-1 PMC8233176

[9] Dahlgren C, Spanberg E, Svereus S, Dackehag M, Wandell P, Rehnberg C. Short- and intermediate-term impact of DTC telemedicine consultations on subsequent healthcare consumption. Eur J Health Econ. 2024;25(1):157–76. 36823408 10.1007/s10198-023-01572-z PMC9950019

[10] Muller S. Electronic medical records: the way forward for primary care research? Fam Pract. 2014;31(2):127–9. 24627543 10.1093/fampra/cmu009 PMC3969524

[11] Wirehn AB, Karlsson HM, Carstensen JM. Estimating disease prevalence using a population-based administrative healthcare database. Scand J Public Health. 2007;35(4):424–31. 17786807 10.1080/14034940701195230

[12] Carlsson AC, Wandell P, Osby U, Zarrinkoub R, Wettermark B, Ljunggren G. High prevalence of diagnosis of diabetes, depression, anxiety, hypertension, asthma and COPD in the total population of stockholm, Sweden - a challenge for public health. BMC Public Health. 2013;13(1):670. 23866784 10.1186/1471-2458-13-670 PMC3724714

[13] Finley CR, Chan DS, Garrison S, Korownyk C, Kolber MR, Campbell S, Eurich DT, Lindblad AJ, Vandermeer B, Allan GM. What are the most common conditions in primary care? Systematic review. Can Fam Physician. 2018;64(11):832–40. 30429181 PMC6234945

[14] Wandell P, Carlsson AC, Wettermark B, Lord G, Cars T, Ljunggren G. Most common diseases diagnosed in primary care in stockholm, sweden, in 2011. Fam Pract. 2013;30(5):506–13. 23825186 10.1093/fampra/cmt033

[15] Burstrom B. Will Swedish healthcare reforms affect equity? BMJ. 2009;339:b4566. 20028781 10.1136/bmj.b4566

[16] Almgren T, Wilhelmsen L, Samuelsson O, Himmelmann A, Rosengren A, Andersson OK. Diabetes in treated hypertension is common and carries a high cardiovascular risk: results from a 28-year follow-up. J Hypertens. 2007;25(6):1311–7. 17563546 10.1097/HJH.0b013e328122dd58

[17] Forslund T, Carlsson AC, Ljunggren G, Arnlov J, Wachtler C. Patterns of Multimorbidity and pharmacotherapy: a total population cross-sectional study. Fam Pract. 2021;38(2):132–40. 32766818 10.1093/fampra/cmaa056 PMC8006765

[18] Ludvigsson JF, Andersson E, Ekbom A, Feychting M, Kim JL, Reuterwall C, Heurgren M, Olausson PO. External review and validation of the Swedish National inpatient register. BMC Public Health. 2011;11:450. 21658213 10.1186/1471-2458-11-450 PMC3142234

[19] Aili K, Campbell P, Michaleff ZA, Strauss VY, Jordan KP, Bremander A, Croft P, Bergman S. Long-term trajectories of chronic musculoskeletal pain: a 21-year prospective cohort latent class analysis. Pain. 2021;162(5):1511–20. 33230006 10.1097/j.pain.0000000000002137 PMC8054552

[20] Larsson C, Hansson EE, Sundquist K, Jakobsson U. Chronic pain in older adults: prevalence, incidence, and risk factors. Scand J Rheumatol. 2017;46(4):317–25. 27885914 10.1080/03009742.2016.1218543

[21] Bondesson E, Larrosa Pardo F, Stigmar K, Ringqvist A, Petersson IF, Joud A, Schelin MEC. Comorbidity between pain and mental illness - Evidence of a bidirectional relationship. Eur J Pain. 2018;22(7):1304–11. 29577509 10.1002/ejp.1218

[22] Försäkringskassan (The Insurance Fund): Report - Follow-up of the development of sickness absence in 2020 (in Swedish: Rapport - Uppföljning av sjukfrånvarons utveckling 2020). In.: Försäkringskassan. 2022:143.

[23] Kostopoulou O, Oudhoff J, Nath R, Delaney BC, Munro CW, Harries C, Holder R. Predictors of diagnostic accuracy and safe management in difficult diagnostic problems in family medicine. Med Decis Making: Int J Soc Med Decis Mak. 2008;28(5):668–80. 10.1177/0272989X08319958 18556634

[24] Hjerpe P, Merlo J, Ohlsson H, Bengtsson Bostrom K, Lindblad U. Validity of registration of ICD codes and prescriptions in a research database in Swedish primary care: a cross-sectional study in Skaraborg primary care database. BMC Med Inf Decis Mak. 2010;10:23. 10.1186/1472-6947-10-23 PMC2864192 20416069

[25] Espinosa-Gonzalez AB, Delaney BC, Marti J, Darzi A. The role of the state in financing and regulating primary care in europe: a taxonomy. Health Policy. 2021;125(2):168–76. 33358033 10.1016/j.healthpol.2020.11.008

[26] Anell A. Choice and privatisation in Swedish primary care. Health Econ Policy Law. 2011;6(4):549–69. 20701829 10.1017/S1744133110000216

[27] Svereus S, Kjellsson G, Rehnberg C. Socioeconomic distribution of GP visits following patient choice reform and differences in reimbursement models: evidence from Sweden. Health Policy. 2018;122(9):949–56. 30144946 10.1016/j.healthpol.2018.07.017

[28] Orueta JF, Urraca J, Berraondo I, Darpon J, Aurrekoetxea JJ. Adjusted clinical groups (ACGs) explain the utilization of primary care in Spain based on information registered in the medical records: a cross-sectional study. Health Policy. 2006;76(1):38–48. 15946763 10.1016/j.healthpol.2005.04.005

[29] Sundquist K, Malmstrom M, Johansson SE, Sundquist J. Care need index, a useful tool for the distribution of primary health care resources. J Epidemiol Community Health. 2003;57(5):347–52. 12700218 10.1136/jech.57.5.347 PMC1732439

[30] Aramrat C, Choksomngam Y, Jiraporncharoen W, Wiwatkunupakarn N, Pinyopornpanish K, Mallinson PAC, Kinra S, Angkurawaranon C. Advancing Multimorbidity management in primary care: a narrative review. Prim Health Care Res Dev. 2022;23:e36. 35775363 10.1017/S1463423622000238 PMC9309754

[31] Wandell PE, Carlsson AC. Gender differences and time trends in incidence and prevalence of type 2 diabetes in Sweden–a model explaining the diabetes epidemic worldwide today? Diabetes Res Clin Pract. 2014;106(3):e90–92. 25451899 10.1016/j.diabres.2014.09.013

[32] Carlsson AC, Li X, Holzmann MJ, Wandell P, Gasevic D, Sundquist J, Sundquist K. Neighbourhood socioeconomic status and coronary heart disease in individuals between 40 and 50 years. Heart. 2016;102(10):775–82. 26864672 10.1136/heartjnl-2015-308784 PMC4846504

[33] Carlsson AC, Wandell PE, Journath G, de Faire U, Hellenius ML. Factors associated with uncontrolled hypertension and cardiovascular risk in hypertensive 60-year-old men and women–a population-based study. Hypertens Research: Official J Japanese Soc Hypertens. 2009;32(9):780–5. 10.1038/hr.2009.94 19557006

[34] Nilsson G, Strender LE. Management of heart failure in primary health care. A retrospective study on electronic patient records in a registered population. Scand J Prim Health Care. 2002;20(3):161–5. 12389753 10.1080/028134302760234618

[35] Wandell PE, de Faire U, Hellenius ML. High intake of alcohol is associated with newly diagnosed diabetes in 60 years old men and women. Nutr Metab Cardiovasc Dis. 2007;17(8):598–608. 16997538 10.1016/j.numecd.2006.05.005

[36] Martin-Merino E, Ruigomez A, Johansson S, Wallander MA, Garcia-Rodriguez LA. Study of a cohort of patients newly diagnosed with depression in general practice: prevalence, incidence, comorbidity, and treatment patterns. Prim Care Companion J Clin Psychiatry. 2010;12(1):PCC08m00764. 10.4088/PCC.08m00764blu PMC2882810 20582294

[37] Dahlgren C, Geary L, Hasselstrom J, Rehnberg C, Schenck-Gustafsson K, Wandell P, Euler MV. Recording a diagnosis of stroke, transient ischaemic attack or myocardial infarction in primary healthcare and the association with dispensation of secondary preventive medication: a registry-based prospective cohort study. BMJ Open. 2017;7(9):e015723. 10.1136/bmjopen-2016-015723 PMC5623465 28939569

