# Supplementary material for: The most common diagnoses in primary care, and changes over time, in the total population of Stockholm, Sweden
Source: BMC Prim Care. 2025 Aug 1;26:235. doi: 10.1186/s12875-025-02938-3 (PMC12315263; doi:10.1186/s12875-025-02938-3)
Supplement: Supplementary file 1 — Supplementary Material 1. [file 12875_2025_2938_MOESM1_ESM.docx]

**Supplementary Table S1. Age distribution in the population in Region Stockholm January 1^st^ 2019, the number of physical visits to physicians in primary care (PC) 2019 and 2021, and number of visits to physicians per capita in primary care (PC) 2019 and 2021.**

|  | **Population 2019**  **(numbers)** | | |  |  | **PC physical visits**  **per capita 2019** | | |  | **PC physical visits**  **per capita 2021** | | |  | **PC digital visits**  **per capita 2021** | | |  | **PC physical and digital visits**  **per capita 2021** | | |
| --- | --- | --- | --- | --- | --- | --- | --- | --- | --- | --- | --- | --- | --- | --- | --- | --- | --- | --- | --- | --- |
| **Age 2019** | **Gender** | |  |  |  | **Gender** | |  |  | **Gender** | |  |  | **Gender** | |  |  | **Gender** | |  |
| **(years)** | **Women** | **Men** | **Total** |  |  | **Women** | **Men** | **Total** |  | **Women** | **Men** | **Total** |  | **Women** | **Men** | **Total** |  | **Women** | **Men** | **Total** |
| **18-44** | 429 175 | 446 143 | 875 318 |  |  | 1.39 | 0.79 | 1.09 |  | 1.03 | 0.60 | 0.81 |  | 0.37 | 0.19 | 0.28 |  | 1.40 | 0.79 | 1.09 |
| **45-64** | 289 466 | 294 875 | 584 341 |  |  | 1.84 | 1.26 | 1.55 |  | 1.43 | 1.02 | 1.22 |  | 0.28 | 0.16 | 0.22 |  | 1.71 | 1.18 | 1.44 |
| **65-79** | 152 707 | 139 098 | 291 805 |  |  | 2.59 | 2.36 | 2.48 |  | 1.96 | 1.63 | 1.80 |  | 0.12 | 0.09 | 0.10 |  | 2.07 | 1.72 | 1.90 |
| **80-w** | 62 633 | 40 247 | 102 880 |  |  | 3.81 | 4.06 | 3.91 |  | 2.11 | 1.95 | 2.05 |  | 0.05 | 0.05 | 0.05 |  | 2.17 | 2.01 | 2.10 |
| **Total** | 933 981 | 920 363 | 1 854 344 |  |  | 1.89 | 1.32 | 1.61 |  | 1.38 | 0.95 | 1.16 |  | 0.28 | 0.16 | 0.22 |  | 1.66 | 1.11 | 1.38 |
